# Local cooling during hot water immersion improves perceptions without inhibiting the acute interleukin-6 response

**DOI:** 10.1007/s00421-021-04616-5

**Published:** 2021-02-28

**Authors:** R. G. Mansfield, S. P. Hoekstra, J. J. Bill, Christof A. Leicht

**Affiliations:** 1 School of Sport, Exercise, and Health Sciences, Loughborough University, Loughborough, UK; 2 The Peter Harrison Centre for Disability Sport, School of Sport, Exercise, and Health Sciences, Loughborough University, Loughborough, UK

**Keywords:** Heat therapy, Inflammatory response, Inflammation, Affect, Comfort

## Abstract

**Purpose:**

Passive elevation of body temperature can induce an acute inflammatory response that has been proposed to be beneficial; however, it can be perceived as uncomfortable*.* Here, we investigate whether local cooling of the upper body during hot water immersion can improve perception without inhibiting the interleukin-6 (IL-6) response.

**Methods:**

Nine healthy male participants (age: 22 ± 1 years, body mass: 83.4 ± 9.4 kg) were immersed up to the waist for three 60-min water immersion conditions: 42 °C hot water immersion (HWI), 42 °C HWI with simultaneous upper-body cooling using a fan (FAN), and 36 °C thermoneutral water immersion (CON). Blood samples to determine IL-6 plasma concentration were collected pre- and post-water immersion; basic affect and thermal comfort were assessed throughout the intervention.

**Results:**

Plasma IL-6 concentration was higher for HWI and FAN when compared with CON (*P* < 0.01) and did not differ between HWI and FAN (*P* = 0.22; pre to post, HWI: 1.0 ± 0.6 to 1.5 ± 0.7 pg·ml^−1^, FAN: 0.7 ± 0.5 to 1.1 ± 0.5 pg·ml^−1^, CON: 0.5 ± 0.2 to 0.5 ± 0.2 pg·ml^−1^). At the end of immersion, basic affect was lowest for HWI (HWI: − 1.8 ± 2.0, FAN: 0.2 ± 1.6, CON 1.0 ± 2.1, *P* < 0.02); thermal comfort for HWI was in the uncomfortable range (3.0 ± 1.0, *P* < 0.01 when compared with FAN and CON), whereas FAN (0.7 ± 0.7) and CON (-0.2 ± 0.7) were in the comfortable range.

**Conclusion:**

Local cooling of the upper body during hot water immersion improves basic affect and thermal comfort without inhibiting the acute IL-6 response.

## Introduction

Heat therapy, the passive exposure of the body to a heat stimulus by means of hot water immersion or sauna, for example, is increasingly gaining attention as a health intervention to improve cardiometabolic health, particularly relevant for populations unable to exercise (Hoekstra et al. 2020). Hot water immersion has the potential to acutely increase plasma interleukin-6 (IL-6) concentration (Laing et al. 2008; Brunt et al. 2018; Hoekstra et al. 2018), which may be one mechanism to explain the downregulation of markers of chronic low-grade inflammation following repeated hot water immersion (Oyama et al. 2013; Brunt et al. 2018; Hoekstra et al. 2018). Whilst the importance of core temperature (*T*_core_) in the induction of inflammatory markers has been identified (Gibson et al. 2016), experimental evidence of upregulated IL-6 in heated isolated myotubes (Welc et al. 2012) implies that passive temperature increases in peripheral muscle might be sufficient to induce this systemic inflammatory response. Indeed, a peripheral heat stimulus that only moderately changes *T*_core_ (~ 0.6 °C) (Kaldur et al. 2016) can also acutely elevate plasma IL-6 concentration.

Despite the promising evidence for heat therapy as a health intervention, hot water immersion can be uncomfortable and can result in a negative perceptual response (Hoekstra et al. 2018) similar to that of high-intensity exercise (Jung et al. 2014; Hoekstra et al. 2017). Hedonic theory states that behaviour is more likely to be repeated if it is associated with pleasure, hence, the affective response for a given activity is associated with adherence to it (Ekkekakis et al. 2008). In the absence of such long-term evidence for heat therapy, it has been shown that affective responses during exercise can predict the adherence to physical activity 6 and 12 months later (Williams et al. 2008). In analogy, adjustments to current passive heating protocols may improve the affective response, and as a result, promote uptake and increase adherence to heat therapy. Naturally, elevations of *T*_core_ and skin temperature (*T*_skin_) are observed during hot water immersion; both have been shown to negatively affect thermal comfort (Frank et al. 1999). However, it has also been demonstrated that thermal comfort during hot water immersion can be improved using short bouts of fanning that decrease *T*_skin_ (Kato et al. 2001), which therefore represents a promising intervention to positively impact perceptions for interventions of a longer duration.

It is important to ensure that such adjustments to existing protocols do not interfere with heat therapy-induced health benefits. It is, therefore, crucial to assess the effectiveness of interventions that may be perceived as less challenging, but more enjoyable, as a reduced challenge might reduce the acute inflammatory response. For example, the acute cytokine response is dependent on the physiological stressor in a dose-dependent manner, supported by evidence obtained during both exercise (Pedersen and Febbraio 2008) and hot water immersion (Oehler et al. 2001; Whitham et al. 2007; Laing et al. 2008; Faulkner et al. 2017; Hoekstra et al. 2018, summarised in Hoekstra et al. 2020). Further, the adrenaline response to an intervention that is perceived as more comfortable may be dampened, adrenaline being a rough measure for the extent of the physiological challenge and a marker of sympathetic activation. This, in turn, might impact the inflammatory response, as adrenaline can independently increase plasma IL-6 concentration (Steensberg et al. 2001). Finally, adjustments to hot water immersion protocols that lead to blunting of the *T*_core_ increase may limit the potential of temperature-related benefits.

Therefore, the primary aim of this study was to investigate the effect of localised upper-body cooling on the perceptual, IL-6 and adrenaline responses to lower-limb hot water immersion. It was hypothesised that localised cooling would reduce perceived heat stress and result in a more positive perceptual response, whilst not reducing the heat stimulus to an extent that would attenuate the acute increase in plasma IL-6 concentration.

## Methods

### Participants

Data of nine healthy males (age: 22 ± 1 years, body mass: 83.4 ± 9.4 kg; height: 180 ± 5 cm, weekly exercise 6.7 ± 3.4 h) were analysed in this study. Participants gave written informed consent after being instructed about the procedures of the study, which were approved by the Local Ethical Committee, in accordance with the Declaration of Helsinki and its later amendments. Data from a tenth participant were discarded as resting IL-6 plasma concentration varied considerably between the three visits (0.58, 3.59, and 6.55 pg·ml^−1^) and exceeded 9 standard deviations of the group mean for the two visits displaying the above-average values. The sample size chosen was based on Hoekstra et al. (2018), who detected a highly significant trial x condition interaction in the acute IL-6 plasma concentration response (effect size 1.71, *P* < 0.001) in a repeated measures design and in conditions similar to the HWI and CON conditions investigated in the current study.

### Procedures

Participants were invited to the laboratory for three visits in randomised order: hot water immersion (HWI; water temperature 42.0 ± 0.2 °C), hot water immersion with simultaneous cooling using a fan (FAN; water temperature 42.0 ± 0.1 °C), and thermoneutral water immersion (CON; water temperature 36.0 ± 0.1 °C). Participants were seated in an upright position immersed up to the belly button for 60 min and were allowed to drink water ad libitum. During FAN, a fan was placed at a distance of 1 m from the participants, pointing at their upper body; wind speed (1.3 ± 0.2 m s^−1^) was measured at head height with a Kestrel 4400 monitor (Nielsen-Kellerman, Boothwyn, PA, US). Immersion was followed by a 60-min recovery period of seated rest in the laboratory. Visits were separated by a minimum of 48 h and were completed within 14 days. Participants refrained from exercise, consumption of alcohol and caffeine, and standardised their diet using a food diary for 24 h before visits. All trials started between 11:00 and 14.30, with the same starting time for each individual to account for a possible circadian rhythm in any of the outcome measures. Ambient air temperature did not differ between conditions (HWI 21.6 ± 0.5 °C, FAN 21.8 ± 0.6 °C, CON 21.3 ± 0.4 °C, *P* = 0.78).

For each visit, upon arrival, participants inserted a rectal probe for the measurement of *T*_core_ (YSI 400 series; YSI, Yellow Springs, US). Skin thermistors (iButton DS1922 L-F5 thermochron data logger, Homechip Ltd, Milton Keynes, UK) were applied at four sites (posterior upper arm, chest, anterior thigh, anterior calf; Choi et al. 1997) for the measurement of *T*_skin_. Height and nude body mass were measured. Pre-immersion measurements were then taken after 30 min of seated rest. At pre- and post-immersion, heart rate (HR) was recorded (Polar RS400; Polar, Kempele, Finland), and systolic and diastolic blood pressure (SBP and DBP; Microlife BP3AC1-1; Microlife, Cambridge, UK) were measured in duplicate on the arm at the level of the heart. On completion of immersion, nude body mass was measured again, and sweat loss was calculated by subtracting the post-body mass from the pre-body mass, taking water consumption into account. Skin temperature was recorded continuously, five-minute averages are reported; *T*_core_ was recorded in 15-min intervals. Mean *T*_skin_ was calculated using the Ramanathan-4 W calculation (Choi et al. 1997), which was then used to calculate heat storage in combination with *T*_core_ (Havenith et al. 1995). Before, immediately upon completion of immersion, and 60 min post immersion venous blood samples were taken by venipuncture.

Perceptions were reported at pre immersion, every 15 min during immersion, and at 30 min and 60 min post immersion. Basic affect was measured using the Feeling Scale (Hardy and Rejeski 1989), for which lower scores indicate decreasing feelings of pleasure and subsequently a more negative affective response. Additional perceptual responses were reported using scales for thermal comfort and thermal sensation (Epstein and Moran 2006). The thermal comfort scale shows satisfaction with the thermal environment from − 4 (very uncomfortable—cold) to + 4 (very uncomfortable—hot), zero being comfortable, whereas the thermal sensation scale reflects the perception of the thermal environment on a scale from 1 (very cold) to 9 (very hot), zero being neutral.

Upon completion of the third visit, participants were given a perception evaluation sheet to indicate their fondness for each condition using a 9-point scale and chose a favourite condition, giving up to 3 reasons explaining their choice.

### Biochemical analyses

Blood was collected in tripotassium EDTA (K_3_EDTA) monovettes. The K_3_EDTA tubes were centrifuged immediately for 10 min at 1500*g* and 4 °C, and plasma was stored at − 80 °C until batch analysis. Plasma IL-6 (high-sensitivity; R&D Systems, Abingdon, UK) and adrenaline (Tecan UK Ltd, Reading, UK) concentrations were determined using enzyme-linked immunosorbent assay (ELISA) kits, according to the manufacturers’ instructions using a microplate reader (Varioskan Flash, ThermoScientific, Waltham, US). Intra-assay coefficients of variation were determined for IL-6 and adrenaline through duplicates analysis and were 4.0% and 6.2% respectively.

### Statistical analyses

All values are given as means ± SD. Normality of data was checked using the Shapiro–Wilk test, and a logarithmic transformation was performed for IL-6 data for which non-normality was detected. A repeated-measures analysis of variance (ANOVA) was then performed for IL-6, adrenaline and body temperature measures, using Bonferroni-corrected post hoc pairwise comparisons. If Mauchly’s test statistic was significant, a Greenhouse–Geisser correction was used. Individual time points were evaluated using repeated measures ANOVAs for normally distributed data (*T*_core_, *T*_skin_, and heat storage). For thigh *T*_skin_, HR, DBP, water consumed, and sweat rate, normality could not be achieved after a logarithmic transformation and related-samples Wilcoxon signed-rank test were performed to compare individual time points between conditions. P values for individual time point analyses are reported without a Bonferroni correction (Perneger 1998). Friedman tests were used to assess perception scale data over time and between conditions. Finally, an exploratory correlations analysis was conducted, investigating bivariate relationships between temperature and thermal perception variables, as well as temperature and plasma markers measured at the end of the immersion period of the HWI trial, reporting Spearman’s Rho. The 25th version of the statistical package SPSS Statistics (SPSS, Chicago, Illinois, US) was used for all analyses; statistical significance was accepted at *P* < 0.05.

## Results

### Inflammatory response

**Fig. 1:**
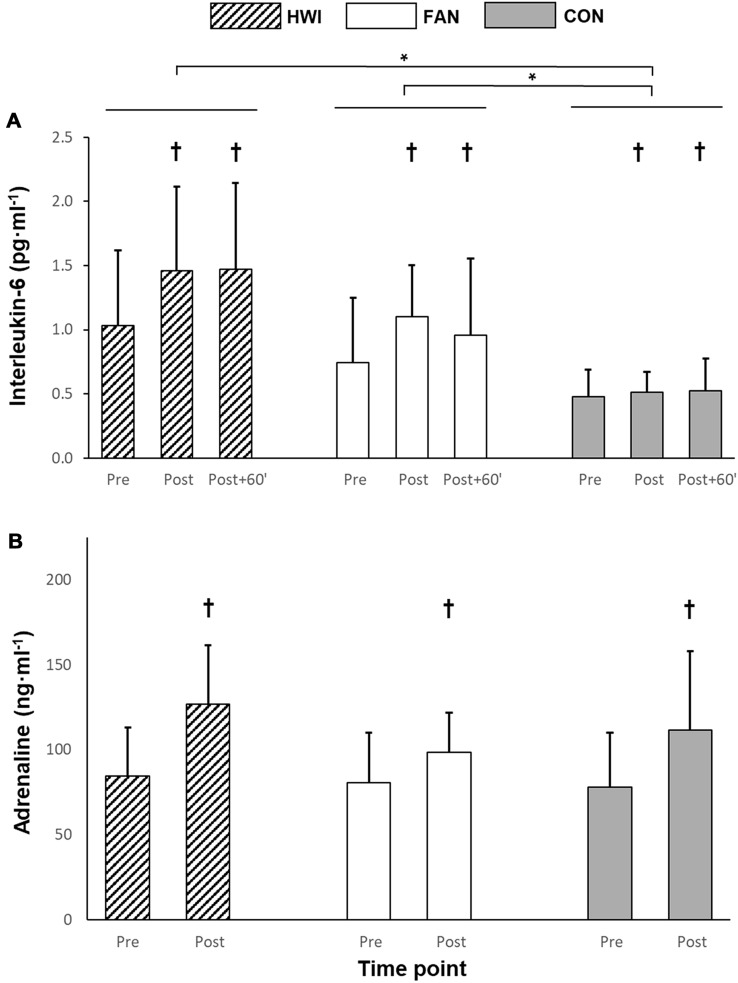
Acute changes in plasma IL-6 (**a**) and adrenaline (**b**) concentration in response to water immersion. HWI, hot water immersion; FAN, hot water immersion with simultaneous cooling using a fan; CON, control condition. Data reported as mean and standard deviation. *Significant difference between conditions. ^†^Significantly higher than Pre (*P* < 0.05)

Plasma IL-6 concentration increased over time (*P* < 0.001). Higher IL-6 concentrations were found for HWI (*P* = 0.006; 95% confidence interval for difference (CI) 0.25–1.39 pg·ml^−1^) and FAN (*P* = 0.003; 95% CI 0.02–0.84 pg·ml^−1^) when compared with CON, however, HWI and FAN did not differ from each other (*P* = 0.22; 95% CI − 0.18–0.95 pg·ml^−1^; Fig. 1a). Plasma adrenaline concentration increased from pre to post (*P* < 0.001; 95% CI 22.9–39.6 ng·ml^−1^), however, no significant effect of condition (*P* = 0.30) or time x condition interaction were found (*P* = 0.31; Fig. 1b).

### Perceptions

For both HWI and FAN, thermal sensation, thermal comfort and basic affect changed over time (*P* < 0.001), which was not the case for CON (*P* > 0.39, Table 1). Thermal sensation differed between all conditions at all time points of immersion; highest scores were reported during HWI, followed by FAN and CON (*P* < 0.05, Table 1). In the first thirty minutes of immersion, thermal comfort scores were higher for HWI when compared with the other conditions (*P* < 0.01), whilst FAN and CON did not differ at 15 min (*P* = 0.16) and at 30 min (*P* = 0.06) of immersion. In the second half of immersion, thermal comfort differed between all conditions (*P* < 0.05), and was in the range of + 3 (“uncomfortably hot”) for HWI, and in the range of − 1 to + 1 (“comfortable”) for FAN and CON. Basic affect was lower for HWI at 30 min, 45 min and 60 min when compared with FAN and CON (*P* < 0.05). During HWI, basic affect was in the range of 3 (“fairly bad”), whilst it was in the range of 0–1 (“neutral” to “fairly good”) during FAN and CON. There were no differences in the affective responses between FAN and CON throughout the immersion period (*P* > 0.06 for all comparisons). None of the perception measures differed in the recovery period (*P* > 0.11 for all comparisons).

**Table 1:** Perceptions in response to water immersion

Parameter	Condition	Time point						
		Pre	15’	30’	45’	60’	Post + 30’	Post + 60’
Thermal sensation 1 (very cold) to 5 (neutral) to 9 (very hot)	HWI*	4.6 ± 0.5	**7.1 ± 0.3**^**††**^	**7.7 ± 0.7**^**†**^	**7.3 ± 0.9**^**†**^	**7.8 ± 0.4**^**††**^	5.1 ± 0.6	4.8 ± 0.4
	FAN*	5.1 ± 0.6	**5.9 ± 0.9**^**†**^	**6.1 ± 0.9**^**†**^	**6.3 ± 0.5**^**†**^	**6.2 ± 0.4**^**††**^	4.8 ± 0.4	4.9 ± 0.3
	CON	4.9 ± 0.6	**5.0 ± 0.7**^**†**^	**4.7 ± 0.7**^**†**^	**4.6 ± 0.9**^**†**^	**4.3 ± 0.7**^**††**^	4.8 ± 0.7	4.8 ± 0.4
Thermal comfort − 4 (very uncomfortable, cold) to Zero (comfortable) to + 4 (very uncomfortable, hot)	HWI*	− 0.1 ± 0.3	**1.9 ± 0.9**^**†**^	**2.4 ± 0.7**^**††**^	**2.6 ± 1.1**^**†**^	**3.0 ± 1.0**^**††**^	0.2 ± 0.4	0.1 ± 0.3
	FAN*	0.0 ± 0.0	0.2 ± 0.4	0.7 ± 0.7	**0.9 ± 0.6**^**†**^	**0.7 ± 0.7**^**†**^	0.0 ± 0.0	0.0 ± 0.0
	CON	− 0.1 ± 0.3	0.0 ± 0.0	0.0 ± 0.3	− **0.1 ± 0.8**^**†**^	− **0.2 ± 0.7**^**†**^	0.0 ± 0.0	0.0 ± 0.0
Basic affect − 5 (negative) to Zero (neutral) to + 5 (positive)	HWI*	1.1 ± 1.7	0.3 ± 1.8	− **1.1 ± 2.3**^**†**^	− **1.4 ± 1.9**^**†**^	− **1.8 ± 2.0**^**†**^	1.2 ± 1.7	1.4 ± 2.1
	FAN*	1.1 ± 1.5	0.8 ± 1.5	0.2 ± 1.5	0.0 ± 1.5	0.2 ± 1.6	1.0 ± 1.4	1.1 ± 1.5
	CON	1.1 ± 1.5	1.1 ± 1.5	0.9 ± 1.7	0.9 ± 1.8	1.0 ± 2.1	1.4 ± 2.1	1.3 ± 1.8

Data reported as mean ± SD

Significant differences are highlighted in bold

*HWI* hot water immersion, *FAN* hot water immersion with simultaneous cooling using a fan, *CON* control condition

*Effect of time (*P* < 0.001), ^†^different from other two conditions (*P* < 0.05)

^††^Different from other two conditions (*P* < 0.01)

Fondness scores were higher for FAN (6.0 ± 1.3) and CON (5.9 ± 1.6) than for HWI (2.4 ± 0.9; *P* < 0.01). There was no difference in fondness between FAN and CON (*P* = 0.90). FAN was selected as favourite condition by *N* = 5, CON by *N* = 4, and HWI by *N* = 0. Qualitative feedback following the intervention to support this choice included: “HWI is too uncomfortable”; “HWI too hot”; “HWI was dreadful”; “FAN is comfortable”; “FAN neutralises hot temperature”; “FAN means you sweat less”; “FAN is much more bearable”, “CON was too cold”; and “CON was slightly cool”.

### Thermophysiology

**Fig. 2:**
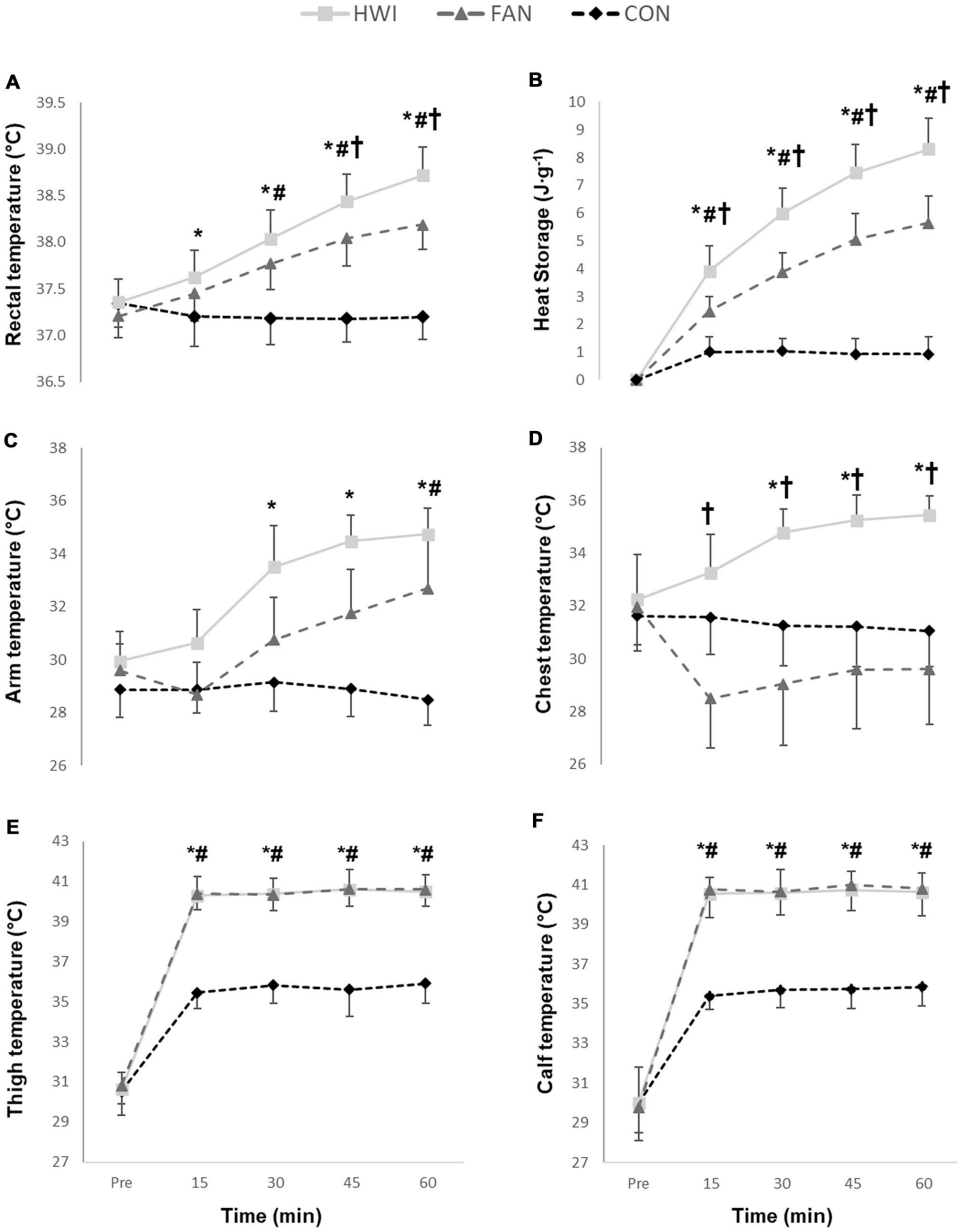
Acute changes in rectal temperature (**a**), heat storage (**b**) and skin temperatures for arm (**c**), chest (**d**), thigh (**e**) and calf (**f**) in response to water immersion. HWI, hot water immersion; FAN, hot water immersion with simultaneous cooling using a fan; CON, control condition. Data reported as mean and standard deviation. Significant difference between *HWI and CON, ^**#**^FAN and CON and ^†^HWI and FAN (*P* < 0.05)

A main effect of time (*P* < 0.001), condition (*P* < 0.001) and a time x condition interaction (*P* < 0.001) indicated a differential *T*_core_ increase between conditions with largest increases observed during HWI, followed by FAN (Fig. 2a). Similarly, a main effect of time (*P* < 0.001), condition (*P* < 0.001), and time × condition interaction (*P* < 0.001) was found for heat storage (Fig. 2b). Arm and chest *T*_skin_ were higher for HWI than for FAN over large parts of the intervention (*P* < 0.05, Fig. 2c, d). In contrast, thigh and calf *T*_skin_ during HWI and FAN did not differ (*P* > 0.48) and were higher than during CON (*P* < 0.05, Fig. 2e, f).

### Bivariate relationships

Core temperature was significantly correlated with basic affect (*R* = 0.68, *P* = 0.046), chest *T*_skin_ was significantly correlated with basic affect (*R* = 0.80, *P* = 0.018) as well as with thermal sensation (*R* = 0.79, *P* = 0.020). All other relationships between perception and temperature variables did not reach significance (*P* > 0.05). Likewise, adrenaline and IL-6 plasma concentrations were not significantly correlated with temperature variables (*P* > 0.05).

### Cardiovascular response and hydration

Heart rate during immersion differed between all conditions (*P* < 0.01); highest values were found during HWI and lowest values during CON. A time × condition interaction (*P* = 0.003) indicated a higher SBP during HWI compared with CON during immersion (*P* = 0.04). A main effect of time (*P* < 0.001) indicated a reduction in DBP during immersion, with no difference between conditions (*P* = 0.07).

Water consumption differed between all conditions (*P* < 0.001), highest values were found during HWI and lowest values during CON. Sweat loss was higher during HWI and FAN when compared with CON (*P* < 0.001). However, sweat loss did not differ between HWI and FAN (*P* = 0.67, Table 2).

**Table 2:** Cardiovascular response and hydration during water immersion

Parameter	HWI		FAN		CON	
	Pre	Immersion	Pre	Immersion	Pre	Immersion
HR (beats∙min^−1^)	66 ± 9	**107 ± 19**^**#**^	66 ± 6	**90 ± 15**^**#†**^	66 ± 12	62 ± 13
SBP (mmHg)	128 ± 7	**135 ± 13**^#^	125 ± 10	125 ± 11	130 ± 13	119 ± 9
DBP (mmHg)*	68 ± 6	57 ± 6	66 ± 5	56 ± 6	70 ± 5	60 ± 6
Water consumed (ml)	N/A	**651 ± 311**^**#**^	N/A	**421 ± 174**^**#†**^	N/A	144 ± 233
Sweat loss (ml)	N/A	**907 ± 258**^**#**^	N/A	**815 ± 325**^**#**^	N/A	63 ± 78

Data reported as mean ± SD

Significant differences are highlighted in bold

*HWI* hot water immersion, *FAN* hot water immersion with simultaneous cooling using a fan, *CON* control condition, *HR* heart rate, *SBP* systolic blood pressure, *DBP* diastolic blood pressure

*Effect of time; significant difference to ^#^CON and ^†^HWI (*P* < 0.05)

No trial order effects were observed for inflammatory responses (*P* > 0.14), perceptions (*P* > 0.08) or thermophysiology (*P* > 0.15).

## Discussion

The main findings of this study were: (1) The local cooling applied during FAN did not reduce the acute IL-6 elevations to passive heat exposure; (2) FAN was associated with improved basic affect, thermal comfort and thermal sensation, indicating a reduction in perceived heat stress compared with HWI; (3) Fondness ratings for FAN were higher when compared with HWI.

### Inflammatory response

Higher rates of evaporative and convective heat loss during FAN reduced overall heat load, resulting in an attenuated *T*_core_ increase during FAN (~ 1.0 °C) when compared with HWI (~ 1.5 °C). Similarly, heat storage during FAN was attenuated (~ 5 J∙g^−1^) compared with HWI (~ 8 J∙g^−1^). However, despite the reduced overall heat load during FAN when compared with HWI, the acute IL-6 response did not differ between HWI and FAN. This may seem counter-intuitive, given that a body of research investigating a range of temperatures and intervention durations implies a dose–response relationship between heat stimulus and the inflammatory response (Oehler et al. 2001; Whitham et al. 2007; Laing et al. 2008; Faulkner et al. 2017; Hoekstra et al. 2018, summarised in Hoekstra et al. 2020). However, whole-body passive hyperthermia experiments that report systemic IL-6 elevations during more modest (~ 0.6 °C) *T*_core_ increases (Kaldur et al. 2016) question whether pronounced *T*_core_ increases are a requirement for an acute IL-6 response. Other lines of enquiry that investigate the acute hyperthermia-induced inflammatory response, therefore, place the focus on peripheral tissue temperature, rather than *T*_core_. For example, muscle tissue is a producer of IL-6 during hyperthermia, as shown by isolated myotubes that increase IL-6 mRNA and protein secretion when heated (Welc et al. 2012). This may have been an important mechanism explaining the systemic IL-6 concentration increase during HWI and FAN, the conditions during which the lower body was immersed in hot water, increasing peripheral tissue temperature. Indeed, the potential role of peripheral tissue temperature in the inflammatory response provided the rationale for the FAN protocol design of the current study, aiming to heat the large muscle mass of the lower extremities to a similar extent as during HWI whilst reducing upper body *T*_skin_.

Lower extremity *T*_skin_ serves as a proxy measure for peripheral tissue temperature, however, we must highlight that deep tissue temperature during FAN was possibly lower than for HWI: previous research documents a higher superficial than deep tissue temperature in the first hour of a HWI intervention (Rodrigues et al. 2020). In addition, in the present study, the lower *T*_core_ during FAN suggests that blood supplying the lower extremities was colder during FAN than HWI, potentially causing some further reductions in lower extremity deep tissue temperature (Raccuglia et al. 2016). Nonetheless, the acute increase in systemic IL-6 concentration was not affected by this possible peripheral tissue cooling. The potential of FAN to mount an inflammatory response, therefore, encourages further investigation of FAN–like protocols in chronic studies, as the repeated acute inflammatory response has been suggested to decrease low-grade inflammation resulting from both chronic exercise training (Gleeson et al. 2011) or regular bouts of passive hyperthermia (heat therapy) (Hoekstra et al. 2020). Indeed, pronounced *T*_core_ elevations during heat therapy may not be a requirement for reductions of markers of chronic low-grade inflammation, as such reductions have been reported following daily hot water immersion at 40 °C for only 10 min (Oyama et al. 2013), a duration unlikely to lead to large elevations of *T*_core_. This again adds to the evidence that with respect to the inflammatory response, increases in *T*_skin_ and peripheral tissue temperature may be more important than *T*_core_ elevations.

We assessed the adrenaline response because the activation of the autonomic nervous system is mechanistically linked to the inflammatory response: adrenaline can independently increase IL-6 concentration (Steensberg et al. 2001), and acute increases in IL-6 plasma concentration are blunted in cervical spinal cord injury, a condition accompanied with autonomic dysfunction (Paulson et al. 2013). Further, thermal clamp experiments show that *T*_core_ impacts the acute increase in the concentration of adrenaline (Rhind et al. 2004), a finding confirmed in hot water immersion studies (Laing et al. 2008; Leicht et al. 2019). Whilst the present study also reports increases in plasma adrenaline concentration, these did not differ between any of the investigated conditions. It is possible that the heat stimulus during HWI and FAN was not large enough to mount a greater adrenaline response than during CON for the healthy young males included in the present study, and that the observed changes are an artefact of the adrenaline circadian rhythm (Åkerstedt and Fröberg 1979). It is also possible that immersion to the waist induces a lower adrenaline response when compared with the aforementioned studies, in which a larger part of the body was immersed (Laing et al. 2008; Leicht et al. 2019). Finally, despite no change in *T*_core_ during CON in the present study, a positive heat storage was found during this condition, which may have contributed to adrenaline concentration elevations following CON. Notably, the finding of an IL-6 response in the absence of an adrenaline response supports previous data in spinal cord injury models, demonstrating that hyperthermia can mount an acute cytokine response despite sympathetic dysfunction (Leicht et al. 2015; Hashizaki et al. 2018). This suggests that the heat stimulus during passive hyperthermia may be a more potent trigger of the IL-6 response than adrenaline. Our findings are further in line with exercise studies demonstrating dramatic elevations in IL-6, but only moderate elevations in adrenaline concentration following long-lasting endurance events (Nieman et al. 2001), pointing out that adrenaline may be of relatively small influence to the IL-6 response in certain contexts.

Summarising the findings on the inflammatory response, the present study demonstrates that HWI and FAN exhibited an altered acute IL-6 response compared with CON, despite a differential *T*_core_ response between trials, and despite no difference in the adrenaline response between trials. Importantly, local cooling did not affect the IL-6 response to passive heating.

### Perceptions

Whilst the IL-6 response to HWI and FAN is comparable to previous studies investigating 60 min of HWI inducing a 1–2 °C *T*_core_ change (Leicht et al. 2015; Faulkner et al. 2017; Hoekstra et al. 2018), the present study further addresses an important issue that has often been overlooked in previous research: perceptions during passive heat stress. The observed effectiveness of a fan to reduce perceived heat strain and the decline in basic affect during passive heating is encouraging for the advancement and promotion of heat therapy. For instance, the affective response during exercise has previously been shown to be a determinant for future exercise participation (Williams et al. 2008). Therefore, FAN-like protocols may lead to better adherence rates in chronic interventions when compared with whole-body forms of passive heating, or they might lead to longer self-selected exposure durations.

Thermal perceptions are strongly influenced by *T*_skin_; local cooling during heat exposure can significantly improve local, and more importantly, whole-body thermal comfort (Kato et al. 2001; Nakamura et al. 2008). This is supported by correlation analyses of the HWI trial of the current study, in which chest *T*_skin_ was found to be correlated to measures of perception. The airflow onto the upper body and the face during FAN is therefore likely a key aspect for the improved perceptions. Indeed, while *T*_skin_ of the immersed part of the body was comparable between HWI and FAN, *T*_skin_ of the exposed upper body was significantly reduced during FAN compared with HWI. Interestingly, sweat loss did not differ between HWI and FAN, implying a higher cooling capacity of the sweat produced during FAN. This is reflected in the qualitative statement “Fan means you sweat less”; whilst not physiologically accurate, the participant likely made this statement as higher rates of sweat evaporation during FAN decreased the sensation of sweat on the skin. Given the relationship between skin wettedness and thermal perceptions (Fukazawa and Havenith 2009) this may have further contributed to the improvements in basic affect and thermal comfort.

Whilst it is debatable to which extent the increases in SBP and HR contributed to the perceived discomfort during HWI, the model of teleoanticipation, linking central output, afferent sensations, and perceptions would support this notion (Hampson et al. 2001). Indeed, drawing on exercise literature, subjective scales such as the Borg scale of perceived exertion have originally been designed as a proxy measure of HR (Borg 1982), and linear relationships between exertion and HR are frequently reported (Borg et al. 1987). More importantly, perhaps, the reduced cardiovascular strain during FAN makes this condition a good alternative to HWI should thermal therapy be considered for at-risk populations, including hypertensive individuals. A further practical consideration rooted in the present findings can be drawn from the net fluid loss following heat exposure. Notably, ad libitum water consumption during FAN was reduced compared with HWI, despite similar sweat rates. This might be explained by the lower levels of heat perceptions during FAN, in turn reducing thirst sensations—a suggestion that has previously been put forward for exercise in cool environments (Maughan et al. 2005). It is important, therefore, to ensure adequate rehydration during heat therapy, especially when simultaneous local cooling is applied, as this appears to reduce the stimulus to drink.

### Future directions

The present study, demonstrating an acute IL-6 response in combination with an improved perceptual response during FAN, provides a solid foundation for future chronic heat therapy studies. Such studies could investigate whether chronic use of FAN-like interventions are as potent as traditional heat therapy interventions (Oyama et al. 2013) to reduce chronic low-grade inflammation, or indeed other aspects of health: it has yet to be determined whether the observed attenuated increase in SBP and HR during FAN reduces or prevents the positive cardiovascular adaptations to hot water immersion as described previously (Brunt et al. 2016). Further, whilst the results indicate improved perceptual responses, it is currently unknown whether these indeed translate to better adherence rates in chronic interventions. Finally, as mentioned in previous reports on heat therapy (Hoekstra et al. 2020), future studies must make an effort to diversify the populations investigated—indeed, some cerebral responses to heat stimuli are sex and age dependent (Chao et al. 2007). In addition, women have been reported to be more sensitive to deviations from an optimally comfortable thermal environment than men (Karjalainen 2012), it is, therefore, possible that the improved perceptual responses seen in the present study may differ between genders. Follow-up investigations should also investigate thermal perceptions in patient populations that may particularly benefit from the health benefits of heat therapy. This is especially pressing in populations in which perceptions might be impacted, for example because of neuronal damage (e.g., spinal cord injury, stroke).

## Conclusion

This study shows that negative perceptual responses during HWI can be attenuated using local cooling without inhibiting the acute inflammatory response. Since perceptual responses may impact long-term adherence, these results can help develop heat therapy protocols that are more likely to be adhered to, which in turn may positively impact chronic disease risk.

## Data Availability

Original data will be made available on request.

## Abbreviations

ANOVA: Analysis of variance
CON: Control condition (thermoneutral water immersion)
DBP: Diastolic blood pressure
FAN: Fan condition (hot water immersion with simultaneous cooling using a fan)
HR: Heart rate
HWI: Hot water immersion condition
IL-6: Interleukin-6
SBP: Systolic blood pressure
*T*_core_: Core temperature
*T*_skin_: Skin temperature
